# *Pyemotes ventricosus* Dermatitis: A Case Report and an Extensive Review of Outbreaks and Clinical Cases

**DOI:** 10.3390/idr14050072

**Published:** 2022-09-05

**Authors:** Iacopo Vellere, Alessandro Di Felice, Annarita Botta, Andrea Angheben, Alessandro Bartoloni, Lorenzo Zammarchi

**Affiliations:** 1 Infectious Disease Unit, Ferrara University Hospital, 44124 Ferrara, Italy; 2 Department of Experimental and Clinical Medicine, University of Florence, 50134 Florence, Italy; 3 Respiratory Infectious Diseases Unit, Cotugno Hospital—A.O.R.N. Dei Colli, 80131 Naples, Italy; 4 Department of Infectious—Tropical Diseases and Microbiology, IRCCS Sacro Cuore Don Calabria Hospital, Negrar Di Valpolicella, 37024 Verona, Italy

**Keywords:** *Pyemotes ventricosus*, parasitic dermatitis, wooden furniture, comet sign, painless bite, self-limiting disease, occupational factors, control measures, agriculture

## Abstract

(1) Background: *Pyemotes ventricosus* dermatitis, caused by free-living mites, could be difficult to diagnose since bites are painless and ectoparasites are not visible. We present an unpublished clinical case that occurred in Italy and an extensive review of clinical cases and outbreaks of *Pyemotes* species. (2) Methods: Case reports and outbreaks of *Pyemotes* spp. were searched for on *Pubmed* and *Embase.* Epidemiological and clinical data were analysed with descriptive statistics. (3) Results: In total, we found 40 case reports and 21 outbreaks to be considered in this review. The majority of cases involved young females, occurred in summer and were observed in Europe. Dermatitis was the most common clinical manifestation. Diagnosis was mainly based on risk factors. Treatment was based on topical steroids and antihistamine drugs. Regarding outbreaks, contact with grain or feed and exposure with infested furniture were the main risk factors. The mean number of involved patients were 69, with symptoms most commonly ending within a week. (4) Conclusions: *Pyemotes ventricosus* dermatitis is underreported, especially in countries like Africa and Central and South-America, since disease is self-limiting and comet sign is reported in a quarter of cases. The reduction in use of pesticides in agriculture could lead to an increased exposure to *Pyemotes* spp. in the future.

## 1. Introduction

*Pyemotes ventricosus* (Newport, 1850) are white to yellow predaceous mites, free-living ectoparasites, which can be barely seen by the naked eye (length: male 0.16 mm; female 0.22 mm; gravid female up to 2.0 mm). They belong to the order Prostigmata, family Pyemotidae. Commonly named “straw itch mite” or “grain itch mite”, they parasite and often kill the larvae of nymphs of many insects which can reside in different products like seed, grain, straw and wood. In particular, Angoumois grain moths (*Sitotroga cerealella*) and common wooden furniture beatles (*Anobium puntactum*) are commonly parasitized [1]. Mites can even be found inside the esoskeletons of dead adult hosts. However, if normal food sources are reduced, *P. ventricosus* may attack horses, cattle, other mammals and humans. Dermatitis can occur if humans come in contact with infested material or when hosts migrate, mature into their adult form or die, so that mites need to find new hosts. Their life cycle occurs internally: they completely develop into the female abdomen, which gives birth to the adult form. Young females and males, as soon as they are extruded, copulate immediately. After mating, females scatter in search of hosts. The growth and development of mites occurs preferably at around 24 °C. Infested furniture should always be treated with ectoparasiticide products, since without a new host mites can survive for up 48 hours. Main risk factor consists in contact with infested products either for occupational reasons or during outdoor or indoor activities. Twenty-four hours after contact with the mite, patients present with a high pruritic central vesicle (at the mite’s bite site), from where a characteristic linear or serpiginous erythematous track, called “comet sign”, can start: this sign is unique and probably due to local lymphangitis [2]. The distribution and morphology of lesions differ from other arthropod bites. Differential diagnosis include chigger bites, another common dermatitis from *Trombicula autumnalis*, which are mites living on vegetation. Chigger bites, usually fewer in number than *P. ventricosus* ones, cause itching papules with a central tiny red spot representing the mite itself: lesions are found in clusters under occlusive clothing (where the mite usually stops and bites), in the groin or around the waist or on the ankle. Occasionally the penis can be severely involved, resulting in summer penile syndrome. 

*P. ventricosus* dermatitis is self-limiting and disappears within 1–3 weeks without treatment. Systemic symptoms are rare, consisting of fever and vomiting. Treatment could be symptomatic, based on oral antihistaminic and topical steroids. Diagnosis can be challenging and is usually based on patient history and clinical signs, since mites are not visible to the naked eye and bites are painless [3]. Starting from a case that occurred in Italy, here we present an extensive review of clinical cases and outbreaks due to the *Pyemotes* species.

## 2. Materials and Methods

We report one case of *P. ventricosus* dermatitis diagnosed in Italy and we present an extensive review of case reports and outbreaks due to the *Pyemotes* spp. Cases and outbreaks were searched for on *Pubmed* and *Embase*. We included only articles written in Italian, English, Spanish, French and German. Epidemiological and clinical data were extracted, retained in two different databases and presented as aggregate in the text through a descriptive statistical analysis. Databases with cases and outbreaks collected and analyzed are presented separately in the Tables S1 and S2 respectively as Supplementary Material.

## 3. Results

### 3.1. Unpublished Case

**Figure 1:**
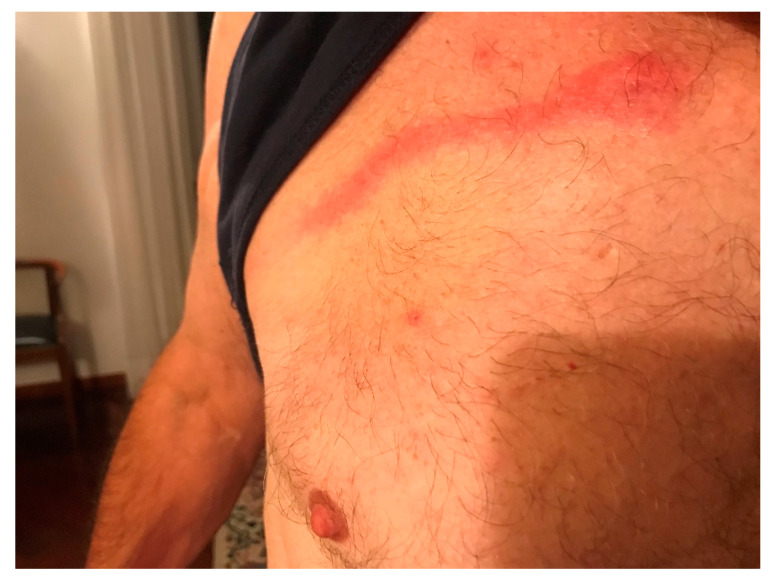
*Pyemotes ventricosus* dermatitis with classical comet sign.

**Figure 2:**
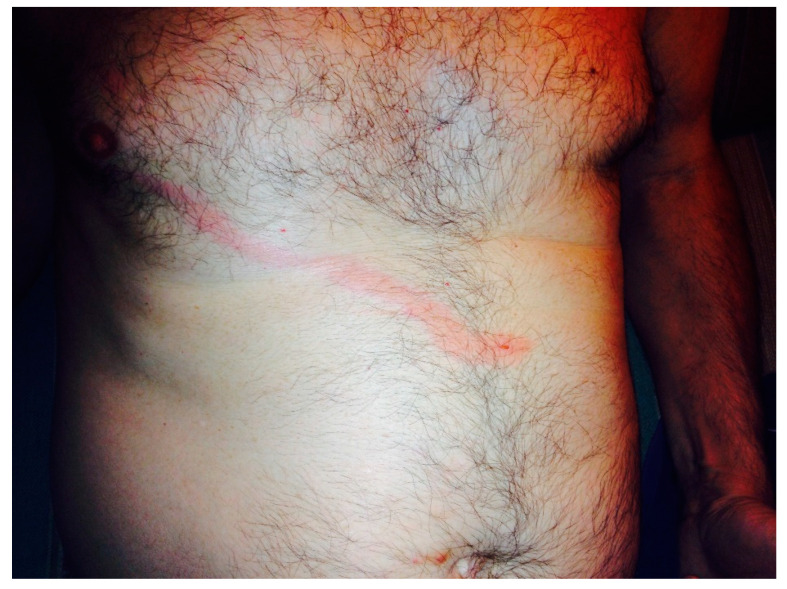
Similar lesion presented six years before.

In September 2020, a 69-year-old Caucasian man with arterial hypertension, for which he had been assuming sartans for years, residing in Central Italy, presented with a pruritic vesicle on his chest, which expanded into a macule and extended laterally with an erythematous track within 48 hours (Figure 1). He used to spend some time in his cellar to repair antique wooden furniture. Six years before, in June 2014, the patient presented a similar lesion, which started from the epigastric region and developed cranially towards the right mammary region (Figure 2). He did not develop any constitutional symptoms. The lesion spontaneously disappeared after ten days. No drug therapy was administered. Based on the characteristics of the lesion, a diagnosis of *P. ventricosus* dermatitis was made.

### 3.2. Review on Cases and Outbreaks Reported

In total, we found 40 case reports and 21 outbreaks to be considered in this review.

Epidemiological and clinical data of case reports are summarized in Table 1. Median age at the diagnosis was 37 years old (IQR 31–48.5). All patients were adults except for a 20-month-old baby [4]. The majority of patients were females (18/32, 56.2%) and most of cases occurred in summer (14/26, 53.8%). Europe was the most represented country of diagnosis (26/40, 65%), followed by US (10/40, 25%). All cases were diagnosed in the Northern Hemisphere. Most cases were caused by *P. ventricosus* (18/31, 58.1%), followed by *P. herfsi* (9/31, 29%) and *P. tritici* (2/31, 6.5%). Regarding risk factors, contact with grain or feed was the most commonly represented (19/40, 47.5%), followed by contact with wooden furniture (14/40, 35%). Cutaneous signs were erythematous papules and vesicles. The unusual presentation of *P. ventricosus* with umbilicated pseudopox-like lesions was also described [5]. The trunk was the most represented site of cutaneous lesions (11/38, 28.9%). Itching dermatitis was always present, whereas systemic signs (mainly low-grade fever) were rarely reported (4/40, 10%). The comet sign, a pathognomonic lesion, was described in around a quarter of cases (11/39, 28.2%). Most cases were diagnosed through clinical signs and the detection of the pathogen in the environment (27/40, 67.5%), whereas only in one case the parasite was microscopically observed after skin scraping [4]. Treatment, consisting of topical steroids and antihistamine drugs, was administered in 17 cases (45.9%).

Epidemiological and clinical data of outbreaks are summarized in Table 2. In particular, we found that all but two (2/21) occurred in the period May–November: however, an outbreak described in January derived from cotton seed in Egypt [6], a region in which the climate is warm throughout the year. Among the identified species (*n* = 31), the most common was *P. ventricosus* (*n* = 18), followed by *P. tritici* (*n* = 3) and *P. herfsi* (*n* = 2); in three cases the mite was not specified. The most recent outbreak was described in Poland in 2017 [7]. Europe was involved in about half (11/21) of the described outbreaks, while the American continent ranks second at 28.6% (6/21). Three outbreaks occurred in Israel [8] and another in Australia [9]. The main risk factor was contact with grain or feed, followed by exposure to infested furniture, a farm environment (e.g., sitting on hay bales) and outdoor activity. Three outbreaks occurred in crews following an infestation of mattresses or wood. The mean number of individuals involved were 69 with the highest number (382 cases) described in the US during a public exhibition [10]. The maximum length of outbreaks was estimated to be 3 years [11] but most of them ended within a 1 week. The characteristics and site of the lesions were the typical ones (erythematous macules-papules with central vesicles localized in back, thorax, limbs, neck and shoulders with pruritus). Admission to hospital for systemic symptoms (fever, vomit, etc.) was reported only in two outbreaks. The most commonly involved specialist was a dermatologist and topical steroids the most commonly administered treatment.

## 4. Discussion

In this article we described one case of *Pyemotes ventricosus* dermatitis and we presented an extensive literature review of case reports and outbreaks due to *Pyemotes* spp.

First outbreak was described in 1909 among the crew of a private yacht due to the contact with infested straw mattresses [12], whereas the last was a case report regarding an Italian woman who spent time in a rural apartment with wooden furniture in 2021 [13]. Throughout more than a century, an evolution in epidemiology can be noted: occupational factors linked to agriculture were predominant in the first outbreaks, whereas nowadays reports of people on summer holiday in the countryside are more frequent. This trend could be due to the more intensive use of pesticides on agriculture products and other control measures like fumigation during the last fifty years [14]. Conversely, the use of pesticides is going to decrease in the XXI century in favor of more sustainable agriculture, as it is included in the UN agenda [15], thus we think that in the future this neglected dermatitis could reappear and become more common.

According to our results, nearly all cases presented in the period May–November, probably because of the mite’s life cycle, activated when temperature reaches 26 °C [16]. Moreover, the two outbreaks described occurring in late autumn and winter were due to imported products from Egypt, which is well known to be a warm country even during winter season [6,17]. All cases except one (Australia) occurred in the Northern Hemisphere. This distribution is probably due to underreporting in countries like Africa and Central and South America, considering that the parasite is ubiquitous and the disease is self-limiting. Interestingly, all patients but one were adults. This age distribution could be explained by the absence of occupational factors during childhood, highlighting the importance of anamnesis for this diagnosis. Diagnosis was mainly based on risk factors, since dermatitis is aspecific and the comet sign is present only in a quarter of cases. An unusual rash (e.g., pseudopox-like) is also possible and virtually every part of the body could be affected. Few cases manifested systemic symptoms, confirming the benign nature of this disease. Treatment was always symptomatic, even if doxycicline was sometimes used. No death from *Pyemotes* dermatitis was reported.

To our knowledge, this is the most recent comprehensive review on *Pyemotes* dermatitis case reports and outbreaks. However, this study presents many limitations due to non-systematic nature and the high number of papers excluded due to lack of data. Moreover, data were analyzed only with descriptive statistics.

## 5. Conclusions

*Pyemotes ventricosus* dermatitis is a benign parasitic dermatitis ubiquitously present but probably underreported in regions such as Africa and Central and South America. Diagnosis is based on suggestive anamnestic data; contact with infested wooden furniture is the main risk factor, whereas occupational factors were predominant in the past. The comet sign, when present, is pathognomonic. Treatment is symptomatic, the lesions are self-limiting but can relapse in case of repeated exposure to an infested environment: treatment of infested forniture with ectoparasiticide products is always needed. Nowadays organic farming plays an essential role in developing a sustainable food system and the reduction in the use of pesticides could lead to increased exposure to *Pyemotes* spp. To conclude, we think that awareness of this disease should be raised not only among dermatologists but also to General Practitioners, pediatricians and other specialists in order not to miss correct diagnosis.

## Figures and Tables

**Table 1:** Table **1.** Epidemiological and clinical characteristics of case reports.

Age	*n* (IQR)
median	37 (31–48.5)
Sex	*n* (%)
Female	18/32 (56.2)
Male	14/32 (43.8)
Not known	8
Region	*n* (%)
Europe	26/40 (65)
Asia	3/40 (7.5)
US	10/40 (25)
Africa	1/40 (2.5)
Season	*n* (%)
Summer	14/26 (53.8)
Autumn	5/26 (19.2)
Spring	6/26 (23.1)
Winter	1/26 (3.8)
Not known	14
Site of cutaneous lesions	*n* (%)
Trunk	11/38 (28.9)
Limb	1/38 (2.6)
Head	1/38 (2.6)
Two or more regions	25/38 (65.9)
Not known	2
Diagnosis	*n* (%)
Clinical	12/40 (30)
Clinical + environmental	27/40 (67.5)
Clinical + biopsy	1/40 (2.5)
Species	*n* (%)
*P. ventricosus*	18/31 (58.1)
*P. herfsi*	9/31 (29.0)
*P. tritici*	2/31 (6.5)
*P. beckeri*	1/31 (3.2)
*P. zwoelferi*	1/31 (3.2)
Not specified	9
Comet sign	*n* (%)
Yes	11/39 (28.2)
No	29/39 (71.8)
Not specified	1
Risk Factors	*n* (%)
Contact with grain or feed	19/40 (47.5)
Contact with wooden furniture	14/40 (35.0)
Other/not specified	7/40 (17.5)
Systemic symptoms	*n* (%)
Yes	4/40 (10.0)
No	36/40 (90.0)
Treatment	*n* (%)
Yes	17/37 (45.9)
No	20/37 (54.1)
Not specified	3

**Table 2:** Epidemiological and clinical characteristics of outbreaks.

Region	*n* (%)
Europe	11/21 (52.4)
Asia	3/21 (14.3)
US	6/21 (28.6)
Oceania	1/21 (4.7)
Period	*n* (%)
May to November	16/18 (88.8)
January	1/18 (5.6)
February	1/18 (5.6)
Not specified	3
Year distribution	*n* (%)
Before 1960	6/21 (28.6)
Between 1960 and 2000	9/21 (42.8)
After 2000	6/21 (28.6)
Subjects involved per outbreak	*n* (%)
<30	10/20 (50.0)
30–50	6/20 (30.0)
>50	4/20 (20.0)
Not specified	1
Diagnosis	*n* (%)
Clinical	1/21 (4.8)
Clinical + environmental	20/21 (95.2)
Species	*n* (%)
*P. ventricosus*	13/18 (72.2)
*P. herfsi*	2/18 (11.1)
*P. tritici*	3/18 (16.7)
Not specified	3
Comet sign	*n* (%)
Yes	2/18 (11.1)
No	16/18 (88.9)
Not specified	3
Risk Factors	*n* (%)
Contact with grain or feed	7/21 (33.3)
Living in same house/city area	6/21 (28.6)
Contact with wooden furniture	3/21 (14.3)
Outdoor activities	2/21 (9.5)
Other	3/21 (14.3)
Systemic symptoms	*n* (%)
Yes	5/21 (23.8)
No/not specified	16/21 (76.2)
Treatment	*n* (%)
yes	10/21 (47.6)
No/not specified	11/21 (52.4)
Admission to hospital	*n* (%)
yes	2/21 (9.5)
No/not specified	19/21 (90.5)

## Data Availability

Not applicable.

## Supplementary Materials

The following supporting information can be downloaded at: https://www.mdpi.com/article/10.3390/idr14050072/s1, Table S1: case reports of *Pyemotes* spp. included in the review; Table S2: outbreaks of *Pyemotes* spp. included in this review.
